# The Effects of Accompanying Ryegrass on Bayberry Trees by Change of Soil Property, Rhizosphere Microbial Community Structure, and Metabolites

**DOI:** 10.3390/plants12213669

**Published:** 2023-10-25

**Authors:** Changxin Li, Gang Li, Xingjiang Qi, Zheping Yu, Yasmine Abdallah, Solabomi Olaitan Ogunyemi, Shuwen Zhang, Haiying Ren, Mohamed Mohany, Salim S. Al-Rejaie, Bin Li, Erming Liu

**Affiliations:** 1 College of Plant Protection, Hunan Agriculture University, Changsha 410128, China; cxli@stu.hunau.edu.cn; 2 State Key Laboratory for Managing Biotic and Chemical Treats to the Quality and Safety of Agro-Products, Institute of Horticulture, Zhejiang Academy of Agricultural Sciences, Hangzhou 310021, China; ligang@zaas.ac.cn (G.L.); qixj@zaas.ac.cn (X.Q.); yuzp@zaas.ac.cn (Z.Y.); zhangsw@zaas.ac.cn (S.Z.); 3 Institute of Biotechnology, Zhejiang University, Hangzhou 310058, China; yasmeen.abdallah@mu.edu.eg (Y.A.); 0622251@zju.edu.cn (S.O.O.); 4 Plant Pathology Department, Faculty of Agriculture, Minia University, Elminya 61519, Egypt; 5 Department of Pharmacology and Toxicology, College of Pharmacy, King Saud University, P.O. Box 55760, Riyadh 11451, Saudi Arabia; mmohany@ksu.edu.sa (M.M.); rejaie@ksu.edu.sa (S.S.A.-R.)

**Keywords:** bayberry, accompanying ryegrass, soil properties, microbial community, metabolites

## Abstract

As a subtropical and tropical tree, bayberry (*Myrica rubra*) is an important fruit tree grown commercially in southern China. Interestingly, our studies found that the fruit quality of bayberry with accompanying ryegrass was significantly improved, but its mechanism remains unclear. The aim of this study was to explore the mechanism of accompanying ryegrass on the beneficial effect of the fruit quality of bayberry by measuring the vegetative growth parameters, fruit parameters with economic impact, physical and chemical properties of rhizosphere soil, microbial community structure, and metabolites of the bayberry with/without ryegrass. Notably, the results revealed a significant difference between bayberry trees with and without accompanying ryegrass in fruit quality parameters, soil physical and chemical properties, microbial community structure, and metabolites. Compared with the control without accompanying ryegrass, the planting of ryegrass increased the titratable sugar, vitamin C, and titratable flavonoid contents of bayberry fruits by 2.26%, 28.45%, and 25.00%, respectively, and decreased the titratable acid contents by 9.04%. Furthermore, based on 16S and ITS amplicon sequencing of soil microflora, the accompanying ryegrass caused a 12.47% increment in *Acidobacteriota* while a 30.04% reduction in *Actinobacteria* was recorded, respectively, when compared with the bayberry trees without ryegrass. Redundancy discriminant analysis of microbial communities and soil properties indicated that the main variables of the bacterial community included available nitrogen, available phosphorus, exchangeable aluminum, and available kalium, while the main variables of the fungal community included exchangeable aluminum, available phosphorus, available kalium, and pH. In addition, the change in microbial community structure was justified by the high correlation analysis between microorganisms and secondary metabolites. Indeed, GC-MS metabolomics analysis showed that planting ryegrass caused a 3.83%–144.36% increase in 19 metabolites such as 1,3-Dipentyl-heptabarbital and carbonic acid 1, respectively, and a 23.78%–51.79% reduction of 5 metabolites compared to the bayberry trees without the accompanying ryegrass. Overall, the results revealed the significant change caused by the planting of ryegrass in the physical and chemical properties, microbiota, and secondary metabolites of the bayberry rhizosphere soils, which provides a new insight for the ecological improvement of bayberry.

## 1. Introduction

Bayberry (*Myrica rubra*) is an important economic fruit that is used as traditional Chinese medicine to extract a variety of antioxidants for anti-inflammatory and anti-allergy purposes [1]. Bayberry contains a variety of flavonoids, such as myricetin, anthocyanins, etc., which have a strong inhibitory effect on cancer cells [2]. Bayberry is widely cultivated in China, which is of great significance in promoting the development of the agricultural economy [3]. In recent years, the policy of planting ryegrass as an accompanying plant in bayberry orchards has been gradually popularized for the reduction of weed reproduction and disease incidence, the increment of soil moisture, and the maintenance of water and fertilizer. Ryegrass has been reported to improve soil structure and the root soil environment [4]. Through our laboratory’s long-term investigation and research on the effects of accompanying ryegrass on bayberry trees, it was found that the fruit quality of bayberry with the planting of ryegrass was improved, which may be mainly due to the reduction of decline disease in bayberry. Although the causal agent of this disease is still unclear, it was proposed that the detrimental effect of this disease on the bayberry plant was mainly due to the change in soil properties, in particular the structure of the soil microbial community.

Scientific and reasonable associated cultivation is one of the most effective means of improving the absorption of mineral nutrients in soil, which invariably improves the absorption of nitrogen, phosphorus, potassium, and manganese in crops, thus promoting crop growth and improving crop yield [5,6]. Plant diversity regulates rhizosphere microbial diversity [7,8]. Recent studies have shown that the intercropping of wheat and hairy vetch with cucumber improved the diversity of the soil microbial community, reduced soilborne diseases, and increased cucumber yield [9]. Also, the rotation of wheat and soybeans with the intercropping of scallion improved the soil microecological environment, increased the diversity of the soil microbial community, and increased the yield of cucumber [10]. In addition, soil metabolites have been reported to be involved in microbiome/plant interactions by regulating growth, development, and stress response processes in many plants [7]. Therefore, it can be inferred that the effects of accompanying ryegrass on bayberry fruit quality may be highly associated with the soil’s physical and chemical properties as well as the soil microbial community and their metabolites.

We hypothesized that the cultivation of ryegrass could impact the soil microbial community and metabolism and drive potential ecological functions in the rhizosphere soil of bayberry. Hence, these effects will be closely related to soil properties, which will cause a reduction in disease incidence and indirectly promote the improvement of bayberry fruit quality. Altogether, in this study, we conducted a three-year experiment with bayberry in the long-term continuous planting of its accompanying plant, ryegrass. Therefore, the aim of this study was to elucidate the underlying mechanism of the impact of accompanying ryegrass on the improvement of the fruit quality of bayberry by comparing the differences in the vegetative growth parameters, fruit parameters with economic impact, soil physical and chemical properties, as well as microbial community structure and metabolites of rhizosphere soils. These findings give new insights into the rhizosphere soil microbial community diversity and metabolite diversity of bayberry with accompanying ryegrass, which provide both a scientific basis for understanding the effects of cultivating accompanying plants on main plants and a microbial insight for sustainable development of the bayberry.

## 2. Results and Discussion

### 2.1. Effects of Accompanying Ryegrass on Vegetative Growth and Fruit Quality of Bayberry

The result from this study indicated that planting ryegrass had no significant effect on the vegetative growth parameters of bayberry trees (Table 1). Indeed, BB-HM displayed 48.60 mm, 2.11 mm, 96.50 mm, 29.10 mm, 0.97 mm, and 43.45 SPAD in twig length, twig diameter, leaf length, leaf width, leaf thickness, and chlorophyll content, respectively, while BB resulted in 46.70 mm, 2.20 mm, 96.80 mm, 29.90 mm, 0.98 mm, and 42.89 SPAD, respectively.

Accompanying ryegrass significantly affected all of the fruit quality parameters except single fruit weight. Indeed, BB-HM resulted in 2.26%, 28.45%, and 25.00% increases in titratable sugar, vitamin C, and titratable flavone, respectively, while it caused a 9.04% decrease in titratable acid compared with the BB (Table 2). In general, the result of this study revealed that BB-HM exhibited a greater effect on improving fruit quality compared to BB. Notably, flavonoids contained in bayberry play an important role in anti-diabetes [11]. Flavonoids include flavonols, flavones, isoflavones, flavanones, flavanols, and chalcones [12]. Hence, it can be inferred that the accompanying ryegrass improved the medicinal value of the bayberry.

### 2.2. Change in Soil Microbial Community Composition

**Figure 1:**
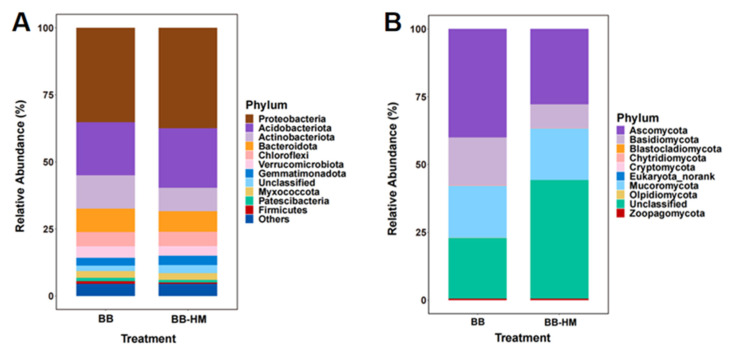
Relative abundance of bacteria (**A**) and fungi (**B**) at the phylum level. BB-HM denotes bayberry and ryegrass co-cultivation; BB indicates bayberry without a cover crop.

The analysis of the soil microbial community shows that *Proteobacteria*, *Acidobacteriota*, and *Actinobacteriota* were the main groups among the top 11 bacterial phyla (Figure 1A). Compared with the BB, the relative abundance of *Actinobacteriota* (*Actinomycetes*) was significantly decreased by 30.04%, while the relative abundance of *Acidobacteriota* was significantly increased by 12.47% in the BB-HM trees. Notably, *Acidothermus cellulolyticus* is capable of producing two xylanases that can efficiently utilize xylans, which are the main components of plant cell walls, for converting plant biomass into products of interest [13]. Therefore, an increase in the relative abundance of *Acidothermus* may improve the biomass utilization capacity of the bayberry trees with ryegrass. Interestingly, protozoa contain a variety of bacterial pathogens [14]. In contrast, *Actinomycetes* are generally beneficial to plants, although some species have been reported to cause plant diseases. Indeed, Actinomycetes serve to protect plants from pathogen infection by producing metabolites such as Jinggangmycin, Wuyimycin, and Zhongshengmycin, while also possessing the potential to promote plant growth through the decomposition and utilization of lignin, cellulose, and organic matter [15,16].

Among the top 10 fungal phylums, *Ascomycota, Mucoromycota,* and *Basidiomycota* were the main phyla, accounting for more than 90% of the fungal sequences (Figure 1B). Compared with BB trees, the relative abundance of *Ascomycota* and *Basidiomycota* in the BB-HM trees was significantly decreased by 30.54% and 50.48%, respectively, while the *unclassified* abundance in the BB-HM trees was significantly increased by 96.59%. Interestingly, *Ascomycota* is known to be a major pathogen of soil-borne diseases and is involved in the breakdown of organic matter and the recycling of nutrients [17]. In contrast, *Basidiomycota* is an important source of biocontrol fungi and plays an important role in plant growth promotion by decomposing soil organic matter, lignin, etc. [18]. These results indicated that there were significant differences in the soil bacterial and fungal community composition at the phylum level of ryegrass and bayberry tree co-cultivation when compared with BB monoculture. Consequently, the composition of the microbial community in the BB-HM bayberry rhizosphere soil was modified due to the presence of ryegrass.

### 2.3. Change in Microbial Community Diversity

**Figure 2:**
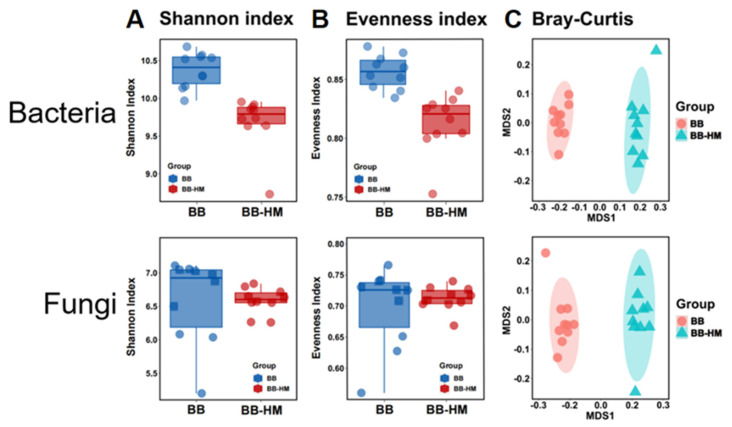
Taxonomic α- and β-diversity estimates from the rhizosphere soil of bayberry trees. (**A**) Shannon indexes of bacteria and fungi; (**B**) evenness indexes of bacteria and fungi; (**C**) PCoA results of soil bacteria and fungi based on OTU abundance. BB-HM denotes bayberry and ryegrass co-cultivation; BB indicates bayberry without a cover crop.

The rhizosphere soil bacterial and fungal community diversity was determined in this study based on 16S rRNA and ITS amplicon sequencing analyses, respectively. Quality-controlled microbial sequence data from 20 bayberry rhizosphere samples (i.e., 3 biological replicates for each treatment) generated a total of 926,740 (16S rRNA gene) and 924,617 (ITS gene) of high-quality sequences. To evaluate the impact of ryegrass presence on the diversity of the microbial community in the rhizosphere soil of bayberry, the Evenness and Shannon indexes were calculated after normalizing the data from each sample to an equal number of reads [19,20]. Bacterial and fungal average read lengths before processing were 415 bp and 238 bp, respectively. To control the differences in sampling effort across plant compartments, we restricted each sample to 30,077 (bacteria) and 29,383 (fungi) sequences per sample before calculating the diversity indexes. The bacterial Shannon indexes and evenness indexes of BB-HM trees were 6.55% and 5.07%, respectively, lower than those of BB trees, while there was no significant difference in the fungal Shannon indexes and evenness indexes between BB-HM and BB trees (Figure 2A,B). Furthermore, bacterial evenness indexes were 1.23 fold greater than those of fungi in BB rhizosphere soil, while bacterial evenness indexes were 1.14 fold greater than those of fungi in BB-HM rhizosphere soil (Figure 2B). Similarly, bacterial Shannon indexes were 1.57 fold greater than those of fungi in BB rhizosphere soil, while bacterial Shannon indexes were 1.47 fold greater than those of fungi in BB-HM rhizosphere soil (Figure 2A). The results indicated that the treatment of accompanying ryegrass significantly affected the microbial diversity and species uniformity of the rhizosphere soil of bayberry.

Furthermore, in order to identify the change in rhizosphere soil community composition of bayberry in the accompanying ryegrass, beta diversity was determined by generating a Bray–Curtis dissimilarity matrix on normalized read abundance data. The effect of accompanying ryegrass on the composition of soil bacteria and fungi was determined by 16S rRNA and ITS amplicon sequencing analysis, respectively, which was carried out from 10 replicates of each treatment by principal coordinates analysis (PCoA) (Figure 2C). Results revealed that both bacterial and fungal microbiomes were well separated between BB and BB-HM bayberries based on the unconstrained principal coordinate analyses (PCoAs), thus suggesting that the microbial community structure varied between BB and BB-HM trees. Hence, the planting of ryegrass resulted in the structural variation of the microbial community of bayberries. Indeed, there was 59.80% total variation in bacterial community structure, while there was a 66.35% total variation in fungal community structure between BB and BB-HM trees based on the result of PCoA.

### 2.4. Soil Properties Related to Microbial Communities

#### 2.4.1. Soil Physical and Chemical Properties

Results from this study indicated that there was a significant change in pH, organic matter, magnesium, available nitrogen, phosphorus, and calcium between BB and BB-HM bayberry rhizosphere soils. Indeed, the pH, organic matter, available nitrogen, phosphorus, kalium, and exchangeable aluminum in BB bayberry rhizosphere soil were 5.34, 72.07%, 169.67 mg/kg, 100.21 mg/kg, 351.79 mg/kg, and 0.14 cmol/kg, respectively (Table 3). However, compared to the BB bayberry, the planting of accompanying ryegrass on bayberry trees resulted in 14.04%, 11.53%, 14.20%, 32.03%, and 27.47% reductions in pH, organic matter, available nitrogen, phosphorus, and kalium, respectively. Notably, a 20.35-fold increase in exchangeable aluminum was observed on the BB-HM.

#### 2.4.2. RDA of Soil Properties and Microbial Communities

**Figure 3:**
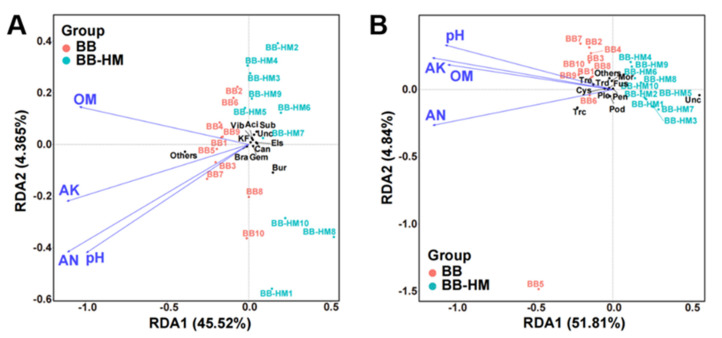
Redundancy discriminant analysis discriminant analysis (RDA) of the rhizosphere bacterial (**A**) and fungal (**B**) community composition at genus level with soil physicochemical properties. Sub: *Subgroup 2_norank*; Bur: *Burkholderia-Caballeronia-Paraburkholderia*; Aci: *Acidobacteriales_norank*; Bra: *Bradyrhizobium*; Vib: *Vibrionimonas*; Els: *Elsterales_norank*; Unc: *Unclassified*; Gem: *Gemmatimonadaceae_uncultured*; KF: *KF-JG30-C25_norank*; Can: *Candidatus Solibacter*; Mor: *Mortierella*; Trc: *Trichocladium*; Tre: *Trechispora*; Trd: *Trichoderma*; Pen: *Penicillium*; Fus: *Fusarium*; Pod: *Podospora*; Ple: *Plectosphaerella*; Cys: *Cystolepiota*; OM: organic matter; AN: available nitrogen; AP: available phosphorus; AK: available kalium; EA: exchangeable aluminum. BB-HM denotes bayberry and ryegrass co-cultivation; BB indicates bayberry without a cover crop.

In this study, the association of soil properties with rhizosphere microbial community composition was examined using a redundancy discriminant analysis (RDA). It was observed that there was a total of 49.89% and 56.65% cumulative variance in the rhizosphere microbial community-factor correction at the bacterial (Figure 3A) and fungal (Figure 3B) genus level, respectively. At the genus level of the bacterial community, the contribution of four main variables was 68.69% of available nitrogen, 66.76% of available phosphorus, 62.33% of exchangeable aluminum, and 61.98% of available kalium. Conversely, at the genus level of the fungal community, the contribution of the four main variables was 93.79% of exchangeable aluminum, 86.20% of available phosphorus, 85.15% of available kalium, and 76.70% of pH explained, respectively. Furthermore, the result revealed that the composition communities of the rhizosphere soil bacteria and fungi were not significantly affected at the genus level by available nitrogen, exchangeable aluminum, available kalium, and available phosphorus. This meant that the composition of bacterial and fungal communities in bayberry rhizosphere soil was not significantly affected by different soil physical and chemical properties.

### 2.5. Change in Rhizosphere Soil Metabolites Composition

Using GC-MS analysis, a total of 84 rhizosphere soil metabolites were detected and identified in all soil samples, including lipids, carbohydrates, acids, alcohols, heterocyclic compounds, etc. Among these compounds, the number of lipids accounted for the greatest, having 21% of the whole metabolite numbers, followed by carbohydrates (13%), acids (13%), and alcohols (11%). These detected compounds, i.e., the soil metabolites, were derived from the plant root exudates, microbial metabolites, and the breakdown of plant, microbe, and soil organic matter [21]. However, the greatest challenge in rhizosphere metabolomics is the ability to distinguish between microbial and plant products [22].

**Figure 4:**
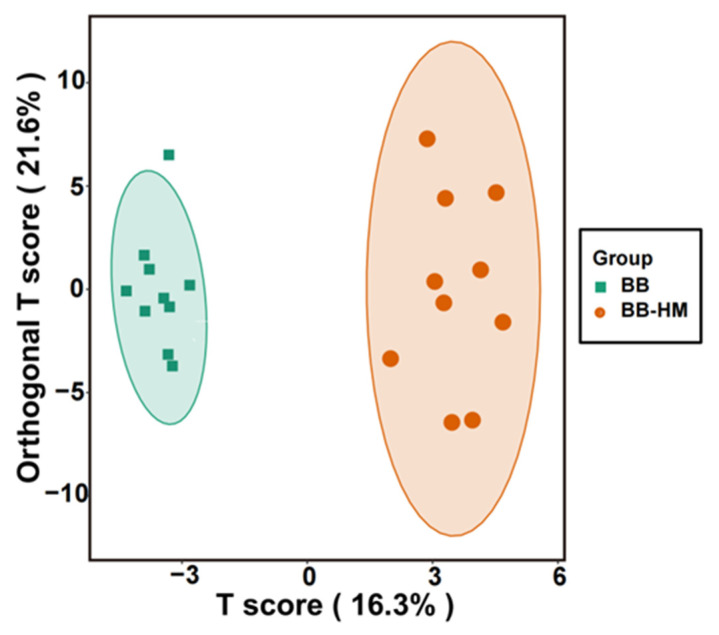
OPLS-DA score map of the rhizosphere soil of bayberry with accompanying ryegrass treatment. BB-HM denotes bayberry and ryegrass co-cultivation; BB indicates bayberry without a cover crop.

Furthermore, a score map of metabolites that can reduce intragroup differences, enlarge intergroup differences, and eliminate the influence of irrelevant factors on the experimental data in order to realize the effective prediction of different samples was successfully constructed based on orthogonal partial least squares-discriminant analysis (OPLS-DA, a supervised statistical method of discriminant analysis). Results showed that the distribution of BB-HM bayberry rhizosphere soils was well separated from that of BB bayberry rhizosphere soils. Indeed, the BB samples (BB) were all distributed in the negative area of t[1] (principal component 1), while the BB-HM samples (BB-HM) were all distributed in the positive area of t[1] (Figure 4). Also, the parameters of the OPLS-DA are R^2^X (cum) = 0.163 and R^2^Y (cum) = 0.216, revealing the interpretability and prediction ability of the BB-BB-HM OPLS-DA model. These results suggest that the metabolite changes in bayberry rhizosphere soils were associated with accompanying ryegrass.

### 2.6. Analysis of Soil Secondary Metabolites Differential Content

**Figure 5:**
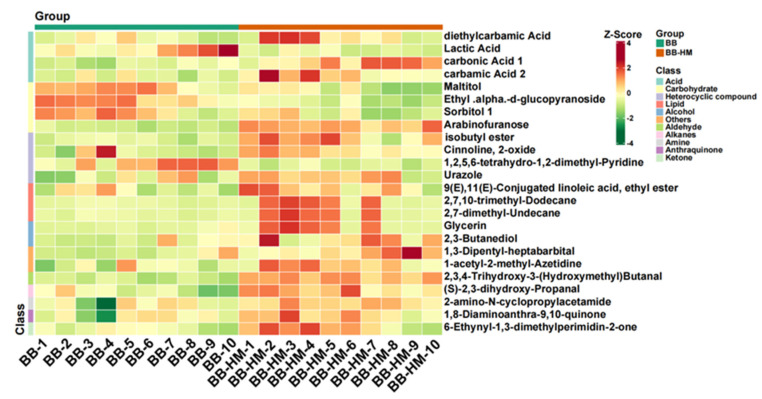
Thermogram analysis of differential metabolites in bayberry rhizosphere soil. BB-HM denotes bayberry and ryegrass co-cultivation; BB indicates bayberry without a cover crop.

In order to observe pattern changes in metabolite content, those with statistically significant differences were normalized, and the resulting clustering heat map was drawn. Here, metabolites with a *p* < 0.05 and a VIP ≥ 1.0 were regarded as significant differential metabolites. The contents of 24 metabolites significantly (*p* < 0.05) differed between the BB and BB-HM bayberry, with a 3.83%–144.36% increment in 19 metabolites and a 23.78%–51.79% reduction in 5 metabolites of the BB-HM bayberry (Figure 5). Results indicated that the main intermediates were significantly different between BB and BB-HM bayberry rhizosphere soils. Indeed, the contents of 1,3-Dipentyl-heptabarbital and carbonic acid 1 in the BB-HM tree rhizosphere soil were 144.36% and 139.92% higher than those of the BB trees, respectively. Conversely, the relative contents of carbohydrate (sorbitol 1, maltitol, etc.) in BB-HM bayberry rhizosphere soil decreased significantly compared with the BB bayberry rhizosphere soil. Notably, a significant reduction in lactic acid content was recorded (Figure 5), probably due to an increase in soil gas permeability by accompanying ryegrass, which reduces bayberry root anaerobic respiration, hence preventing its rot [23]. Hence, the results suggested that planting accompanying ryegrass may increase soil permeability, reducing bayberry root anaerobic respiration and thereby preventing its rot.

### 2.7. Correlation Analysis of Soil Microorganisms and Metabolites

**Figure 6:**
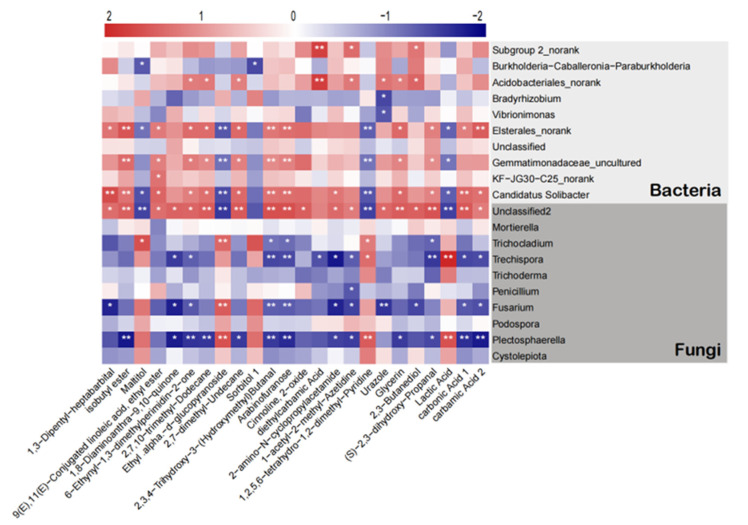
Heatmap correlation analysis between the microorganism relative abundance at the genus level and the metabolite relative contents. * and ** indicates a significant correlation at *p* < 0.05 and *p* < 0.01, respectively. The depth of the red and blue blocks represents the magnitude of the correlation coefficient, where the deep red color implies greater positive correlation while the deep blue implies greater negative correlation. The heatmap correlation between metabolites of bayberry and bacterial and fungi genera was generated using the Spearman correlation coefficient.

This result revealed a correlation at the genus level between microbial groups and secondary metabolites in bayberry rhizosphere soil (Figure 6). Maltitol positively correlated with *Trichocladium* and negatively correlated with *Burkholderia*—Caballeronia—*Paraburkholderia*, *Elsterales_norank*, and *Candidatus Solibacter*. The Ethyl-alpha—D—glucopyranoside positively correlated with *Trichocladium, Fusarium*, and *Plectosphaerella* and negatively correlated with *Elsterales_norank, Gemmatimonadaceae_uncultured,* and *Candidatus Solibacter*. There was a positive correlation between 1,2,5,6-tetrahydro-1,2-dimethyl-Pyridine and *Trichocladium, Trechispora,* and *Plectosphaerella*, while a negative correlation was observed between *Elsterales_norank, Gemmatimonadaceae_uncultured,* and *Candidatus Solibacter*. Lactic Acid positively correlated with *Trechispora* and *Plectosphaerella* and negatively correlated with *Elsterales_norank, Gemmatimonadaceae_uncultured,* and *Candidatus Solibacter*. The other 20 metabolites except Sorbitol 1 were all positively correlated with *Elsterales_norank, Gemmatimonadaceae_uncultured,* and *Candidatus Solibacter*, and negatively correlated with *Trechispora, Fusarium,* and *Plectosphaerella,* respectively.

On the other hand, *Subgroup 2_norank* and *Acidobacteriales_norank* were positively correlated with diethylcarbamic acid. *Elsterales_norank* positively correlated with isobutyl ester, 2,3,4-Trihydroxy-3-(Hydroxymethyl)Butanal, Arabinofuranose, and carbamic Acid 2, and negatively correlated with Ethyl .alpha.-d-glucopyranoside and 1,2,5,6-tetrahydro−1,2-dimethyl-Pyridine. *Gemmatimonadaceae_uncultured* positively correlated with isobutyl ester, 2,3,4-Trihydroxy-3-(Hydroxymethyl)Butanal and Arabinofuranose, and negatively correlated with Ethyl .alpha.-d-glucopyranoside and 1,2,5,6-tetrahydro−1,2-dimethyl-Pyridine. There was a positive correlation between *Candidatus solibacter* with 1,3-Dipentyl-heptabarbital, isobutyl ester, 2,3,4-Trihydroxy-3-(Hydroxymethyl)Butanal, Arabinofuranose, and carbonic acid 1, and a negative correlation was observed with Ethyl .alpha.-d-glucopyranoside and 1,2,5,6-tetrahydro-1,2-dimethyl-Pyridine. *Trichocladium* is positively correlated with Ethyl .alpha.-d-glucopyranoside. A positive correlation was recorded between *Trechispora* and Lactic Acid, while a negative correlation was observed with 2,3,4-Trihydroxy-3-(Hydroxymethyl)Butanal, Arabinofuranose, and (S)-2,3-dihydroxy-Propanal. *Fusarium* is positively correlated with Ethyl .alpha.-d-glucopyranoside, and negatively correlated with 2,3,4-Trihydroxy-3-(Hydroxymethyl)Butanal, Arabinofuranose, and Urazole. Also, *Plectosphaerella* positively correlated with Ethyl .alpha.-d-glucopyranoside, 1,2,5,6-tetrahydro-1,2-dimethyl-Pyridine, and Lactic Acid, and negatively correlated with isobutyl ester, 6-Ethynyl-1,3-dimethylperimidin-2-one, 2,7,10-trimethyl-Dodecane, 2,3,4-Trihydroxy-3-(Hydroxymethyl)Butanal, Arabinofuranose, carbonic Acid 1, and carbamic Acid 2. *Gemmatimonadaceae_uncultured* and *Plectosphaerella* are likely to be the core microbes mediating microorganisms and metabolites (Figure 6).

## 3. Materials and Methods

### 3.1. Field Trial and Sampling

In this study, samples were collected from a field experiment of 15-year-old bayberry (cv. Dongkui) trees in Chun’an County, Hangzhou City, Zhejiang Province. The distance between planting rows was about 4 m × 5 m, with the orchard being managed routinely in the traditional manner. The field trial was established in September 2019, with the accompanying ryegrass being cultivated continuously for three years. In the treatment group BB-HM, bayberry trees were represented with accompanying ryegrass, while in the control group BB, bayberry trees were represented without accompanying ryegrass. In the treatment group, BB-HM, ryegrass was cultivated in the soil below the canopy of bayberry trees, and its effect on bayberry trees was evaluated in December 2022 by measuring substantial variations in bayberry’s vegetative growth parameters, fruit economic characters, microbial community structure, physical and chemical properties, and the metabolomics of rhizosphere soils.

### 3.2. Measurement of Vegetative Growth Parameters

During the fruit maturation period, after approximately 8 months of fertilizer application, twenty branches from each treatment were randomly selected. The diameter of these branches was measured using a digital Vernier caliper (Shanghai Daoju, Shanghai, China). The measurement of chlorophyll content (SPAD) was carried out using a SPAD-502 Plus chlorophyll meter (Minolta, Osaka, Japan). For leaf measurements, the fourth to eighth leaves below the top of the vegetative branches in the middle part of the tree periphery were chosen and sampled. Leaf length (measured from the top to the base of the petiole) and leaf width (measured at the widest point) were determined using a ruler. The leaf thickness was measured using a digital Vernier caliper. Each parameter measured had six repetitions, and the average value was calculated.

### 3.3. Measurement of Fruit Parameters with Economic Impact

During the fruit maturation period, 200 ripe fruits were randomly selected from each treatment. Immediately, the weight of individual fruit was determined, and the samples were stored at −20 °C for later analysis of titratable acid and vitamin C content. An electronic balance (Shanghai Precision Instrument) was used to weigh 15 fruits from each treatment, and the average value was calculated. Titratable sugar and titratable flavone flavonol were measured by the Molecular Device SpectraMax ABS Plus. Titratable acid was determined by acid-base titration [24], and vitamin C was determined by 2–6 dichloroindophenol titration [25].

### 3.4. Soil Sample Collection

In December 2022, at the postharvest time of the fruits, rhizosphere soil samples were collected at the drip line (1.5 m distance) around the crown of the bayberry trees with/without accompanying ryegrass from trees with stable vegetative and reproductive growth. Each replicate consisted of a single tree, and each treatment contained 10 trees with similar vegetative and reproductive growth. The straight-line distance between each treatment was at least 100 m. The soil samples were collected 5 to 10 cm below the surface. Rhizosphere soil was strictly defined as soil particles adhering to the roots. Using the quartering method, about 2 kg of mixed soil samples were collected from each tree under investigation and passed through a 0.45 mm sieve. Half of the soil samples were kept in a refrigerator at −80 °C, and the other half were dried at room temperature for measuring the physical and chemical properties of the soil.

### 3.5. Soil Genome Sequencing

Genomic DNA of soil samples was extracted by the E.Z.N.A.^®^ Soil DNA Kit (Omega Bio-Tek, Norcross, GA, USA). Bacterial diversity was determined by amplifying the 16S rRNA V3-V4 region using primers 341F-5′-CCTAYGGGRBGCASCAG-3′ and 806R-5′-GGACTACNNGGGTATCTAAT-3′. Fungal diversity was determined by amplifying the ITS1 and ITS2 regions using the primers ITS1F-5′-CTTGGTCATTTAGGAAGTAA-3′ and ITS2R-5′-GCTGCGTTCTTCATCGATGC-3′. Genome sequencing was carried out at Shanghai BIOZERON Biotechnology Co., Ltd. (Shanghai, China) using the Illumina PE250 platform. Usearch software (V 10) was used to classify OTUs according to 97% similarity, while the SILVA rRNA database and the UNITE database were used to annotate the representative sequences and the database [26].

### 3.6. Soil Physical and Chemical Properties

After air-drying naturally, the physical and chemical properties of the soil were examined. The pH was determined by a pH meter (the ratio of soil to water was 1:2.5); organic matter was determined by K_2_Cr_2_O_7_ oxidation by external heating method [27]; available N was determined by the Modified Kjeldahl method; available P was determined by hydrochloric acid ammonium fluoride extraction molybdenum antimony anti colorimetry [28]; available kalium was extracted by ammonium acetate; and contents were determined by an ice3500 atomic absorption spectrophotometer [29].

### 3.7. Gas Chromatography-Mass Spectrometry (GC-MS) Metabolomics Analysis

Fresh soil was harvested, weighted, immediately frozen in liquid nitrogen, and stored at −80 °C until needed. Samples were freeze-dried and ground to a powder at room temperature. Approximately 0.5 g of the sample was weighed, followed by the addition of 1 mL of methanol, isopropanol, and water (3:3:2; *v*/*v*/*v*) extract, vortexed for 3 min, and ultrasound for 20 min. The extracts were then centrifuged at 12,000 r/min at 4 °C for 3 min. The supernatant was carefully transferred into a sample vial, and 0.020 mL of internal standard (10 μg/mL) was added and left to evaporate under nitrogen flow. The evaporated samples were transferred to the lyophilizer for freeze-drying. The residue was saved and used for further derivatization.

The derivatization method was as follows: the sample was mixed with a 0.1 mL solution of methoxyamine hydrochloride in pyridine (0.015 g/mL). The mixture was then incubated at 37 °C for 2 h, followed by the addition of 0.1 mL of BSTFA (with 1% TMCS) into the mixture, and kept at 37 °C for 30 min after vortex-mixing. Approximately 0.2 mL of the derivatization solution was pipetted, and n-hexane was added to dilute to 1 mL, which was then filtered with a 0.22 μm organic phase syringe filter, stored in a refrigerator at −20 °C, and tested on the machine within 24 h.

An Agilent 8890 gas chromatograph coupled to a 5977B mass spectrometer with a DB-5MS column (30 m length × 0.25 mm i.d. × 0.25 μm film thickness, J&W Scientific, USA) was employed for GC-MS analysis of soil. Helium was used as a carrier gas at a flow rate of 1.2 mL/min. Injections were made in the front inlet mode with a split ratio of 5:1, and the injection volume was 1 μL. The oven temperature was held at 40 °C for 1 min, which was then raised to 100 °C at 20 °C/min, and finally raised to 300 °C at 15 °C/min, and held at 300 °C for 5 min. All samples were analyzed in scan mode. The ion source and transfer line temperatures were 230 °C and 280 °C, respectively. The determination of metabolite content in bayberry rhizosphere soil was performed by gas chromatography-mass spectrometry (GC-MS) at Metware Biotechnology Co., Ltd. (Wuhan, China).

### 3.8. Statistical Analysis

Community histograms and redundancy discriminant analysis (RDA) were performed using R (V 3.6.2). OPLS-DA was made by R (V 3.6.2) ropls. The heatmap was constructed using the heatmap package in R (V 3.6.2) with pheatmap package. The α-diversity index (Shannon, evenness index) was calculated by Mothur software (V 1.30.1). Excel 2010 was used for preliminary data processing; SPSS 17.0 software was used for the significance test and Kruskal–Wallis test, respectively [30].

## 4. Conclusions

Results of this study revealed that bayberry rhizosphere soil microflora was significantly changed by accompanying ryegrass, which caused an increase in the relative abundance of *Proteobacteria* and *Acidobacteriota* and a decrease in the relative abundance of *Actinobacteriota* compared with the BB. Furthermore, RDA of microbial communities and soil properties showed that four main variables of the bacterial community included pH, organic matter, available phosphorus, and nitrogen, while four main variables of the fungal community included pH, organic matter, available phosphorus, and calcium. In addition, the change in microbial community structure was justified by the high correlation between metabolites and microorganisms at the genus level. Indeed, GC-MS metabolomics analysis showed that the contents of 1,3-Dipentyl-heptabarbital and carbonic acid 1 were increased by 144.36% and 139.92% in BB-HM compared to that of BB, respectively, while the contents of carbohydrate (sorbitol 1, maltitol, etc.) and acid (lactic acid) in BB-HM were lowered compared to that of BB. Overall, the result of this study revealed the significant change caused by accompanying ryegrass in the microbiota, physical and chemical properties, and metabolites of the rhizosphere soils of bayberry, which provides a new insight for the ecological improvement of bayberry.

## Figures and Tables

**Table 1:** Effects of accompanying ryegrass on bayberry vegetative growth parameters.

Parameters	Value	Parameters	Value
Twig length (mm)		Twig diameter (mm)	
BB-HM	48.60 ± 8.16	BB-HM	2.11 ± 0.32
BB	46.70 ± 9.45	BB	2.20 ± 0.39
Leaf length (mm)		Leaf width (mm)	
BB-HM	96.50 ± 0.92	BB-HM	29.10 ± 0.25
BB	96.80 ± 1.03	BB	29.90 ± 0.36
Leaf thickness (mm)		Chlorophyll/(SPAD)	
BB-HM	0.97 ± 0.03	BB-HM	43.45 ± 2.69
BB	0.98 ± 0.06	BB	42.89 ± 3.71

No statistically significant difference was observed. BB-HM denotes bayberry and ryegrass co-cultivation; BB indicates bayberry without a cover crop.

**Table 2:** Effects of accompanying ryegrass on quality parameters of bayberry fruit.

Parameters	Value	Parameters	Value
Single fruit weight (g)		Titratable sugar (mg/g)	
BB-HM	47.08 ± 3.40	BB-HM	109.83 ± 0.41 *
BB	48.95 ± 4.01	BB	107.40 ± 0.42
Vitamin C (mg/100 g)		Titratable acid (%)	
BB-HM	9.66 ± 0.54 *	BB-HM	0.97 ± 0.08
BB	7.52 ± 1.05	BB	1.06 ± 0.07 *
Titratable flavone (mg/g)			
BB-HM	1.60 ± 0.01 *		
BB	1.28 ± 0.01		

* indicates statistically significant differences between experimental conditions (*p* < 0.05). BB-HM denotes bayberry and ryegrass co-cultivation, BB indicates bayberry without a cover crop.

**Table 3:** The pH, physical, and chemical properties of bayberry rhizosphere soil with/without accompanying ryegrass.

Parameters	Value	Parameters	Value
pH		Available phosphorus (mg/kg)	
BB-HM	4.59 ± 0.13	BB-HM	68.11 ± 1.35
BB	5.34 ± 0.19 *	BB	100.21 ± 4.50 *
Organic matter (%)		Exchangeable aluminum (cmol/kg)	
BB-HM	63.76 ± 1.35	BB-HM	2.99 ± 0.30 *
BB	72.07 ± 3.19 *	BB	0.14 ± 0.02
Available nitrogen (mg/kg)		Available kalium (mg/kg)	
BB-HM	145.58 ± 3.58	BB-HM	255.17 ± 8.38
BB	169.67 ± 8.40 *	BB	351.79 ± 19.70 *

BB-HM denotes bayberry and ryegrass co-cultivation, BB indicates bayberry without cover crop. * indicates statistically significant differences between experimental conditions (*p* < 0.05).

## Data Availability

Raw sequence data reported in this paper have been deposited (PRJCA019488) in the Genome Sequence Archive in the BIG Data Center62, Chinese Academy of Sciences, under accession codes CRA019659 for bacterial 16S rRNA gene and fungal ITS gene sequencing data that are publicly accessible at http://bigd.big.ac.cn/gsa (accessed on 4 September 2023).
